# CRISPR-induced exon skipping is dependent on premature termination codon mutations

**DOI:** 10.1186/s13059-018-1532-z

**Published:** 2018-10-17

**Authors:** Tingting Sui, Yuning Song, Zhiquan Liu, Mao Chen, Jichao Deng, Yuanyuan Xu, Liangxue Lai, Zhanjun Li

**Affiliations:** 1 Jilin Provincial Key Laboratory of Animal Embryo Engineering, Key Laboratory of Zoonosis Research, Ministry of Education, Institute of Zoonosis, Jilin University, Changchun, 130062 China; 2 Key Laboratory of Regenerative Biology, Guangzhou Institutes of Biomedicine and Health, Chinese Academy of Sciences, Guangzhou, 510530 Guangdong China

**Keywords:** Exon skipping, PTC, CRISPR/Cas9, Rabbits

## Abstract

In previous studies, CRISPR/Cas9 was shown to induce unexpected exon skipping; however, the mechanism by which this phenomenon is triggered is controversial. By analyzing 22 gene-edited rabbit lines generated using CRISPR/Cas9, we provide evidence of exon skipping at high frequency in premature termination codon-mutated rabbits but not in the rabbits with a premature termination codon mutation in exon 1 rabbits with non-frameshift or missense mutations. Our results suggest that CRISPR-mediated exon skipping depends on premature termination codon mutation-induced nonsense-associated altered splicing.

**Electronic supplementary material:**

The online version of this article (10.1186/s13059-018-1532-z) contains supplementary material, which is available to authorized users.

## Background

The CRISPR/Cas9 system has been widely used to disrupt gene function and to create premature termination codon (PTC), non-frameshift or missense mutations in target genes [1]. Alternatively, cytidine base editors (CBEs), which are composed of a cytidine deaminase fused to Cas9 nickase, can be used to efficiently inactivate genes by precisely converting four codons (CAA, CAG, CGA and TGG) into stop codons [2, 3].

PTCs are commonly caused by frameshift mutations or nonsense mutations, leading to the generation of truncated proteins with deleterious dominant-negative or gain-of-function effects [4, 5]. To reduce these harmful effects, nonsense-mediated decay (NMD), a well-characterized mRNA surveillance system, recognizes and rapidly degrades mRNAs carrying PTC mutations [6–8]. It is also possible for nonsense-associated alternative splicing (NAS) to produce a transcript that no longer contains the PTC [7, 9]. If the spliced transcript has an in-frame mutation, NAS could produce a truncated protein that potentially retains the function of the corresponding full-length protein [10, 11]. Therefore, NMD and NAS can be regarded as yin and yang responses, respectively. Although the NAS response can upregulate alternatively spliced transcripts to skip offending PTC mutations, the exact mechanism by which this effect is triggered remains controversial.

Recently, several studies have reported a high frequency of exon skipping in gene-edited cells and mice generated using the CRISPR/Cas9 system [12–14]. Here, based on summarized analyses of gene-edited rabbits that have been successfully generated in our group [15–19], we present the first evidence that CRISPR-induced exon skipping depends on PTC mutations.

## Results

To investigate whether CRISPR/Cas9-mediated genome editing induces unexpected exon skipping, 22 mutant rabbit lines generated using CRISPR/Cas9 or CBEs were utilized in this study (Table 1). We grouped these rabbits as follows: (1) the D3, L2, K2 and K3 lines, each of which had a non-frameshift mutation, (2) the T2-T6 lines, each of which had a missense mutation, (3) the D2*,* L3, A2, G1, G2, G3, G4, B3 and B4 lines, each of which had a PTC mutation and (4) the M2, M3, Y2 and Y3 lines, each of which carried a PTC in the first exon. Then, genotypes and exon skipping were determined by PCR and RT-PCR, respectively.

**Table 1:** Summary of the CRISPR-induced exon skipping in rabbits

Mutation type	Rabbits lines	Nature of mutation	Predicted ESS	PTC in exon	Exon skipping	Method
Non-frameshift	D3 (Additional file 2: Figure S1)	− 75 bp in exon 51	Yes	No	No	CRISPR/Cas9
	L2 (Additional file 2: Figure S2)	(− 4, + 1) bp in exon 2	Yes	No	No	CRISPR/Cas9
	K2 (Additional file 2: Figure S6)	WT/− 6 bp in exon 3	Yes	No	No	CRISPR/Cas9
	K3 (Additional file 2: Figure S6)	− 6 bp in exon 3	Yes	No	No	CRISPR/Cas9
Missense	T2 (Additional file 2: Figure S7)	C>T in exon 14	No	No	No	BE3
	T3 (Additional file 2: Figure S7)	C>T in exon 14	No	No	No	BE3
	T4 (Additional file 2: Figure S7)	C>T in exon 14	No	No	No	BE3
	T5 (Additional file 2: Figure S7)	C>T in exon 14	No	No	No	BE3
	T6 (Additional file 2: Figure S7)	C>T in exon 14	No	No	No	BE3
PTCs	D2 (Additional file 2: Figure S1)	− 157 bp/− 70 bp in exon 51	Yes	Exon 51	Exon 51 (233 bp)	CRISPR/Cas9
	L3 (Additional file 2: Figure S2)	− 106 bp/− 14 bp in exon 2	Yes	Exon 2	Exon 2 (157 bp)	CRISPR/Cas9
	A2 (Additional file 2: Figure S3)	− 10 bp/− 13 bp in exon 12	Yes	Exon 12	Exon 12 (223 bp)	CRISPR/Cas9
	G1 (Additional file 2: Figure S4)	− 91 bp in exon 5	Yes	Exon 5	Exon 5 (130 bp)	CRISPR/Cas9
	G2 (Additional file 2: Figure S4)	− 14 bp in exon 5	Yes	Exon 5	Exon 5 (130 bp)	CRISPR/Cas9
	G3 (Additional file 2: Figure S4)	− 5 bp/− 91 bp in exon 5	Yes	Exon 5	Exon 5 (130 bp)	CRISPR/Cas9
	G4 (Additional file 2: Figure S4)	− 5 bp in exon 5	Yes	Exon 5	Exon 5(130 bp)	CRISPR/Cas9
	B3 (Additional file 2: Figure S5)	C>T in exon 20	Yes	Exon 20	Exon 20 (242 bp)	BE3
	B4 (Additional file 2: Figure S5)	C>T in exon 20	Yes	Exon 20	Exon 20 (242 bp)	BE3
PTCs in exon 1	M2 (Additional file 2: Figure S8)	C>T in exon 1	No	Exon 1	No	BE3
	M3 (Additional file 2: Figure S8)	C>Tin exon 1	No	Exon 1	No	BE3
	Y2 (Additional file 2: Figure S9)	C>T in exon 1	No	Exon 1	No	BE3
	Y3 (Additional file 2: Figure S9)	C>T in exon 1	No	Exon 1	No	BE3

The ESS sites were predicted by online tool (http://astlab.tau.ac.il/index.php)

−, deletion; +, insert; BE3, Cytidine base editors; ESS, essential splice site

D1, D2, D3, the *DMD* gene-edited rabbits by CRISPR/Cas9; L1, L2, L3, the *LMNA* gene-edited rabbits by CRISPR/Cas9; K1, K2, K3, the *GCK* gene-edited rabbits by CRISPR/Cas9; A1, A2, the *ANO5* gene-edited rabbits by CRISPR/Cas9; G1, G2, G3, G4, G5, the *GHR* gene-edited rabbits by CRISPR/Cas9; B1, B2, B3, B4, the *DMD* gene-edited rabbits by BE3; T1, T2, T3, T4, T5, T6, the *TIA1* gene-edited rabbits by BE3; M1, M2, M3, the *MSTN* gene-edited rabbits by BE3; Y1, Y2, Y3, the *TYR* gene-edited rabbits by BE3

**Fig. 1:**
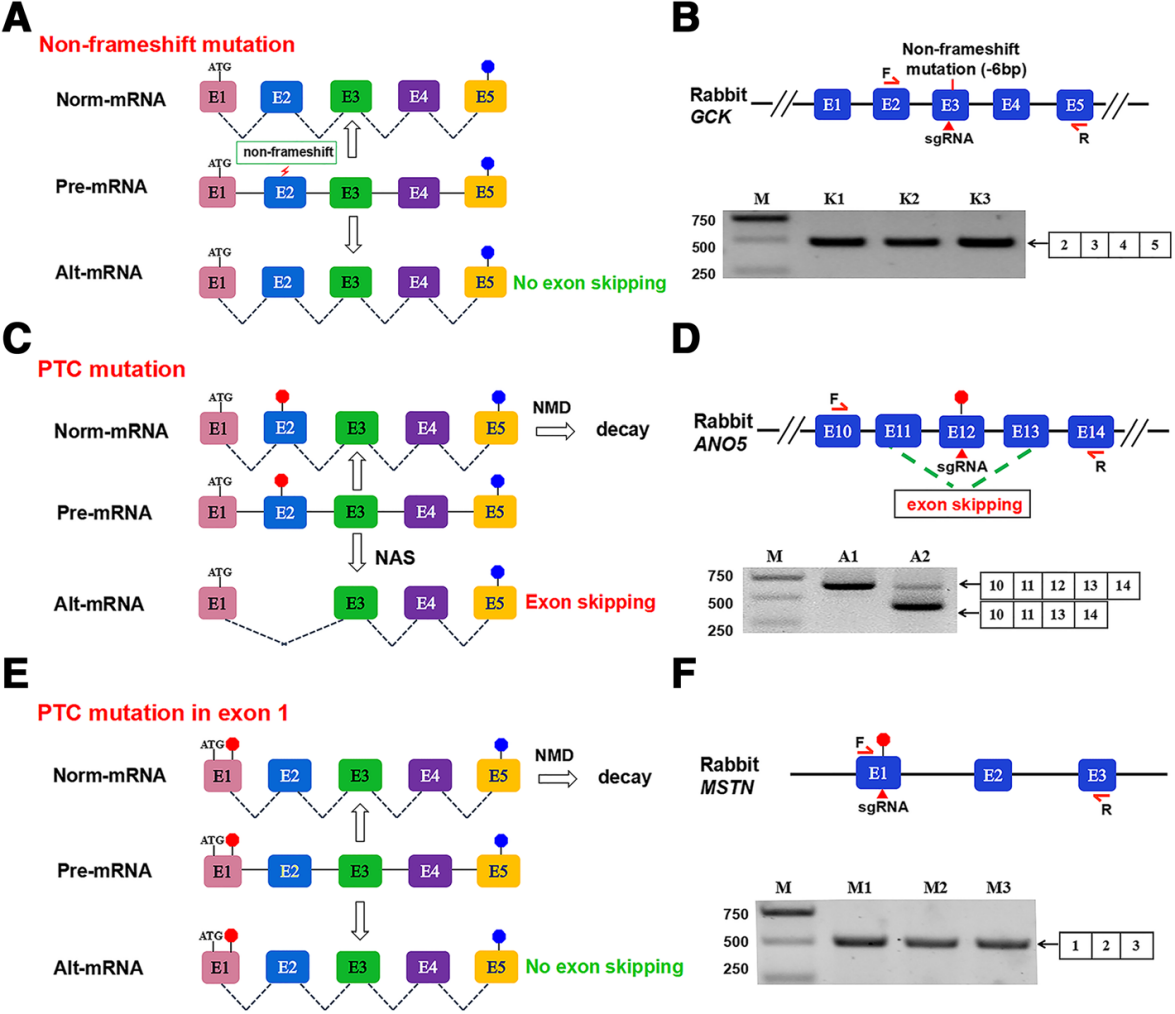
Exon skipping induced using the CRISPR/Cas9 system. **a** No exon skipping in rabbits with a non-frameshift mutation. **b** Non-frame shift mutation in exon 6 of *GCK* did not induce exon skipping. Schematic diagram of sgRNA target site in exon 3 of the rabbit *GCK* gene locus and RT-PCR analysis of *GCK* gene-editing rabbits for exons 2, 3, 4 and 5. Gel images have been cropped. M, which shows the DL2000 ladder, indicates band size. K1, K2, K3, the *GCK* gene-edited rabbits used in this study. **c** CRISPR-mediated exon skipping depends on PTC mutation-induced nonsense-associated altered splicing (NAS). **d** PTC mutation in exon 12 of *ANO5* gene induces exon skipping. Schematic of sgRNA target site in exon 12 of the rabbit *ANO5* gene and RT-PCR analysis of *ANO5* gene-editing rabbits for exons 10, 11, 12, 13 and 14. Gel images have been cropped. M, which shows the DL2000 ladder, indicates band size. A1-A2, the *ANO5* gene-edited rabbits used in this study. **e** No exon skipping in mutated rabbits with a PTC in exon 1. Rectangle, exon; blue octagon, normal stop codon; red octagon, PTC; NMD, nonsense-mediated decay; NAS, nonsense-associated alternative splicing; ATG, initiation codon; E1-E5, different exons. (F) PTCs mutation in exon 1 of MSTN gene did not induce exon skipping. Schematic diagram of sgRNA target site in exon 1 of the rabbit *MSTN* gene locus and RT-PCR analysis of *MSTN* gene editing rabbits for exons 1, 2 and 3. Gel images have been cropped. M, which shows the DL2000 ladder, indicates band size. M1, M2, M3, the *MSTN* gene-edited rabbits used in this study

 It has been shown that CRISPR/Cas9-mediated genome editing induces unexpected alternative splicing or in-frame exon skipping [12, 20]. Thus, in an attempt to determine whether the gene-edited rabbit lines carrying non-frameshift indels or missense mutations exhibited exon skipping, the D3, L2, K2 and K3 rabbits with non-frameshift mutations and the T2-T6 rabbits carrying missense mutations were analysed (Table 1) (Additional file 1). The RT-PCR results showed clear products that corresponded to normal mRNA transcripts (D3, Additional file 2: Figure S1D; L2, Additional file 2: Figure S2D; K2-K3, Additional file 2: Figure S6D; T2-T6, Additional file 2: Figure S7D), suggesting that no exon skipping occurred in the rabbits with non-frameshift and missense mutations (Fig. 1a).

Previous studies have shown that NAS-induced exon skipping is a putative corrective response to skip an offending PTC during pre-mRNA splicing [7, 21]. In addition, exon skipping induced by nonsense mutations has been widely reported in clinical cases [11]. To determine whether PTC mutations induced unexpected exon skipping in mutant rabbit lines, the D2, L3, A2, G1, G2, G3, G4, B3 and B4 rabbit lines were analysed (Table 1). The RT-PCR results showed two products in these rabbits: one product corresponding to normal mRNA (norm-mRNA) and the other product corresponding to alt-mRNA produced via exon skipping (D2, Additional file 2: Figure S1D; L3, Additional file 2: Figure S2D; A2, Additional file 2: Figure S3D; G1-G4, Additional file 2: Figure S4D; B3 and B4, Additional file 2: Figure S5D). These results were also confirmed via Sanger sequencing (D2, Additional file 2: Figure S1E; L3, Additional file 2: Figure S2E; A2, Additional file 2: Figure S3E; G1-G4, Additional file 2: Figure S5E), suggesting that exon skipping occurred in the PTC-mutated rabbits (Fig. 1c).

In addition, we also confirmed this hypothesis at the cellular level. As shown in Additional file 2: Figure S10, specific exon skipping occurred when exons contained a PTC but not when exons contained other types of mutations produced when T in PTC was replaced by G or A. Furthermore, published reports on CRISPR/Cas9-induced exon skipping and clinical case reports have confirmed this principle, which could be used to predict exon skipping induced by the CRISPR/Cas9 system (Additional file 3: Table S1).

To further explore the idea of whether a PTC in exon 1 can induce exon skipping in mutant rabbits, M2, M3, Y1 and Y2 rabbits were analysed. As shown in Additional file 2: Figure S8E and Figure S9E, RT-PCR results clearly showed products corresponding to normal transcripts in these rabbits. Furthermore, qPCR results showed no significant differences in transcript level between exon 1 and other exons (M2-M3, Additional file 2: Figure S8F; Y2-Y3, Additional file 2: Figure S9F), suggesting that no exon skipping was observed in mutated rabbits with a PTC in exon 1 (Fig. 1e).

## Discussion

In this study, we demonstrated that CRISPR-mediated exon skipping depends on PTC mutations but was not observed in rabbits with non-frameshift and missense mutations; these findings are consistent with clinical cases in which mutations induced exon skipping of the *COL11A2* and *FBN1* genes in patients [12, 22, 23]. We also show that this principle can be used to predict CRISPR-mediated exon skipping in future studies. Moreover, our data support the hypothesis of a nuclear scanning mechanism that identifies pre-mRNAs harboring a PTC and then induces exon skipping [7, 24], an idea consistent with observations of unregulated alternative splicing to skip disruption of the reading frame in genes with nonsense mutations [25, 26].

Currently, although the roles of NMD and cis-acting regulatory elements have been proposed to be associated with alternative splicing of exons or exon deletion, the exact mechanisms by which these events are triggered remain elusive. Previous studies have demonstrated that mutations in putative essential splice site (ESS) could cause aberrant or alternative splicing [27–29]. In our study, although ESRs were found in both the non-frameshift mutation lines (D3, L2, K2 and K3) and the PTC-mutated rabbit lines (D2, L3, A2, G1, G2, G3, G4, B3 and B4), exon skipping was identified only in PTC-mutated rabbits. Hence, we speculate that PTC mutations that disrupt ESS may be responsible for exon skipping, although more types of gene mutations and examinations of mechanisms for triggering NAS should be assessed in further research.

## Conclusions

Overall, this article is the first report describing a high frequency of exon skipping in PTC-mutated rabbits generated using the CRISPR/Cas9 system. Moreover, our results highlight that predictions of potential exon skipping are important for interpreting negative results or conducting genotype-to-phenotype studies in genome-edited animals generated using the CRISPR/Cas9 system.

## Additional files

Additional file 1: Supplemental materials and Methods. (PDF 614 kb)

Additional file 2: **Figure S1.** PTC mutation in exon 51 of *DMD* gene induces exon skipping. **Figure S2.** PTC mutation in exon 2 of *LMNA* gene induces exon skipping. **Figure S3.** PTC mutation in exon 12 of *ANO5* gene induces exon skipping. **Figure S4.** PTC mutation in exon 5 of *GHR* gene induces exon skipping. **Figure S5.** PTC mutation in exon 20 of *DMD* gene induces exon skipping. **Figure S6.** Non-frame shift mutation in exon 6 of *GCK* did not induce exon skipping. **Figure S7.** Missense mutations in the last exon of the *TIA1* gene did not induce exon skipping. **Figure S8.** PTCs mutation in exon 1 of *MSTN* gene did not induce exon skipping. **Figure S9.** PTCs in exon 1 of *TYR* gene did not induce exon skipping. **Figure S10.** PTC mutation in exon 2 of *OXT* gene induces exon skipping. (PDF 1853 kb)

Additional file 3: **Table S1.** Examples according to the published reports on CRISPR/Cas9-induce exon-skipping and the case reports in clinical. **Table S2.** PCR primers for genotyping of the gene editing rabbits. **Table S3.** Primers for RT-PCR analysis of exon skipping. **Table S4.** Primers for qPCR analysis. (PDF 561 kb)

## Abbreviations

CBEs: Cytidine base editors
ESS: Essential splice site
indels: Insertions or deletions
NAS: Nonsense-associated alternative splicing
NMD: Nonsense-mediated decay
PTC: Premature termination codon
sgRNA: Single-guide RNA
