# Supplementary material for: CRISPR-induced exon skipping is dependent on premature termination codon mutations
Source: Genome Biol. 2018 Oct 17;19:164. doi: 10.1186/s13059-018-1532-z (PMC6193291; doi:10.1186/s13059-018-1532-z)
Supplement: Supplementary file 1 — Supplemental materials and Methods. (PDF 614 kb) [file 13059_2018_1532_MOESM1_ESM.pdf]

Methods

Genotyping of the gene editing rabbits

Genomic DNA of the gene editing rabbits was extracted and performed to PCR detection as previously study [1]. The sgRNA target sites primers were listed in **Additional File 3: Table S2**.

Exon skipping detection

The RT-PCR and qPCR were performed as previously study [2]. The splicing pattern of mRNA was determined by RT-PCR using primer pairs located different exons of the sgRNA targeted exons. qPCR was used for detected the transcript levels of different exons using exon specific primer. Primers used for RT-PCR and qPCR were shown in **Additional File 3: Table S3 and S4**.

Plasmids construction

To determine whether exon skipping occur in transient system when T in PTC replaced by G and A, the site-specified mutation vector were constructed. Construct *OXT*-C is the WT rabbit oxytocin (*OXT)* gene, which containing 3 exons (NC_013672.1). Construct *OXT*-T contains a PTC mutation (C-T, Q89Stop), construct *OXT*-A contains a missense mutation (C-A, Q89K), and construct *OXT*-G contains a missense mutation (C-G, Q89E). The DNA sequences (*OXT*-C, *OXT*-T, *OXT*-A and *OXT*-G, Fig. S10A and B) were synthesized and cloned into pcDNA3.1 vector by Genscript (Nanjing, China).

Cell culture and transfection

Human kidney epithelial cell line (HEK293T) were cultured in Dulbecco's modified Eagle's medium (DMEM) supplemented with 10% fetal bovine serum (HyClone), and incubated at 37 ℃ in an atmosphere of 5% CO2. The cells were seeded into 6-well plates and transfected using Lipofectamine^{TM} 3000 Reagent (Thermo Fisher Scientific) according to the manufacturer's instructions. After 44 hours, the cells were collected and used for genotyping and exon skipping determination. PCR and RT-PCR primers were listed in **Additional File 3**: **Table S2 and S3.**

Statistical analysis

Data were statistically analysed with GraphPad prism software (T test), and p < 0.05 was considered statistically significant. *$p < 0.05$, **$p < 0.01$, ***$p < 0.001$, **** $p < 0.0001$.

References

1. Sui T, Yuan L, Liu H, Chen M, Deng J, Wang Y, Li Z, Lai L: **CRISPR/Cas9-mediated mutation of PHEX in rabbit recapitulates human X-linked hypophosphatemia (XLH).** *Hum Mol Genet* 2016, **25:**2661-2671.
2. Lv Q, Yuan L, Song Y, Sui T, Li Z, Lai L: **D-repeat in the XIST gene is required for X chromosome inactivation.** *RNA Biol* 2016, **13:**172-176.
