# Supplementary material for: CRISPR-induced exon skipping is dependent on premature termination codon mutations
Source: Genome Biol. 2018 Oct 17;19:164. doi: 10.1186/s13059-018-1532-z (PMC6193291; doi:10.1186/s13059-018-1532-z)
Supplement: Supplementary file 2 — Figure S1. PTC mutation in exon 51 of DMD gene induces exon skipping. Figure S2. PTC mutation in exon 2 of LMNA gene induces exon skipping. Figure S3. PTC mutation in exon 12 of ANO5 gene induces exon skipping. Figure S4. PTC mutation in exon 5 of GHR gene induces exon skipping. Figure S5. PTC mutation in exon 20 of DMD gene induces exon skipping. Figure S6. Non-frame shift mutation in exon 6 of GCK did not induce exon skipping. Figure S7. Missense mutations in the last exon of the TIA1 gene did not induce exon skipping. Figure S8. PTCs mutation in exon 1 of MSTN gene did not induce exon skipping. Figure S9. PTCs in exon 1 of TYR gene did not induce exon skipping. Figure S10. PTC mutation in exon 2 of OXT gene induces exon skipping. (PDF 1853 kb) [file 13059_2018_1532_MOESM2_ESM.pdf]

Supplemental Figures and Figure legends

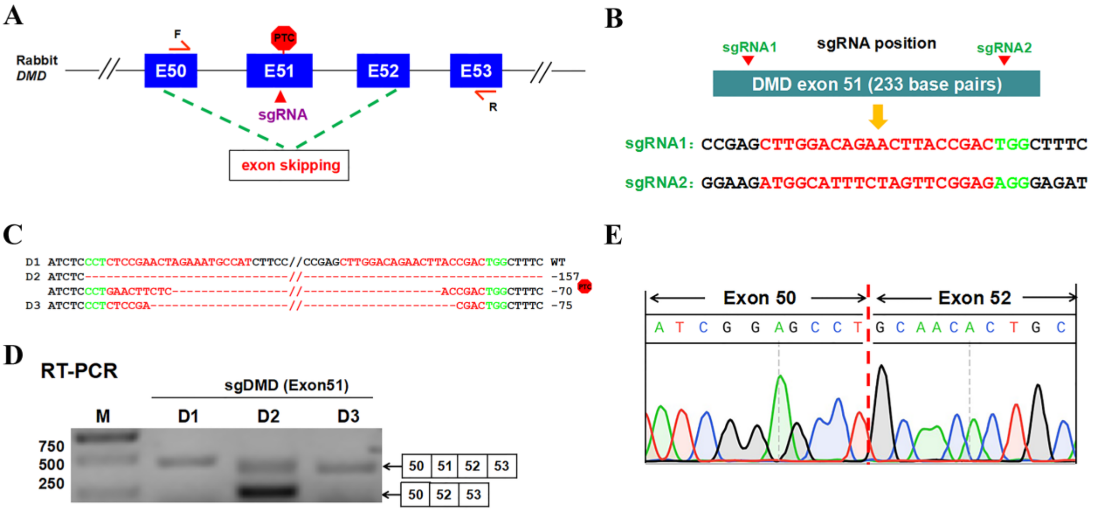

Figure S1: PTC mutation in exon 51 of *DMD* gene induces exon skipping. (A) Schematic diagram of two sgRNA target sites in exon 51 of the rabbit *DMD* locus. *DMD* exons are indicated by the blue boxes; the target site of sgRNAs is indicated by the red triangle; the PTC site is indicated by the red octagon. (B) sgRNA target sites in *DMD* exon 51. (C) Sanger sequencing results for the *DMD* PCR product. (D) RT-PCR analysis of *DMD* gene-editing rabbits for exons 50, 51, 52 and 53. Gel images have been cropped. M, which shows the DL2000 ladder, indicates band size. D1, D2, D3, the *DMD* gene-edited rabbits used in this study. (E) Sanger sequence analysis of cDNA bands confirmed the splicing of exon 51.

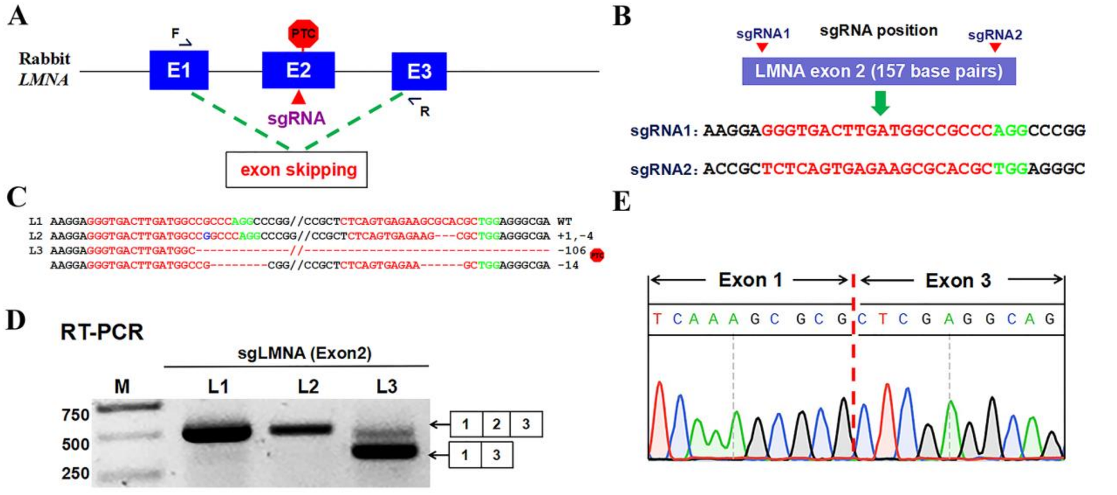

Figure S2: PTC mutation in exon 2 of *LMNA* gene induces exon skipping. (A) Schematic diagram of sgRNA target site in exon 2 of the rabbit *LMNA* locus. *LMNA* exons are indicated by blue boxes; the target site of sgRNAs is indicated in red triangle; the PTC site is indicated by red

octagon. (B) sgRNA target sites in *LMNA* exon 2. (C) Sanger sequencing results of the *LMNA* PCR product. (D) RT-PCR analysis of *LMNA* gene-editing rabbit for exons 1, 2 and 3. Gel images have been cropped. M, which shows the DL2000 ladder, indicates band size. L1, L2, L3, the *LMNA* gene-edited rabbits used in this study. (E) Sequence analysis of cDNA bands confirmed the splicing of exon 2.

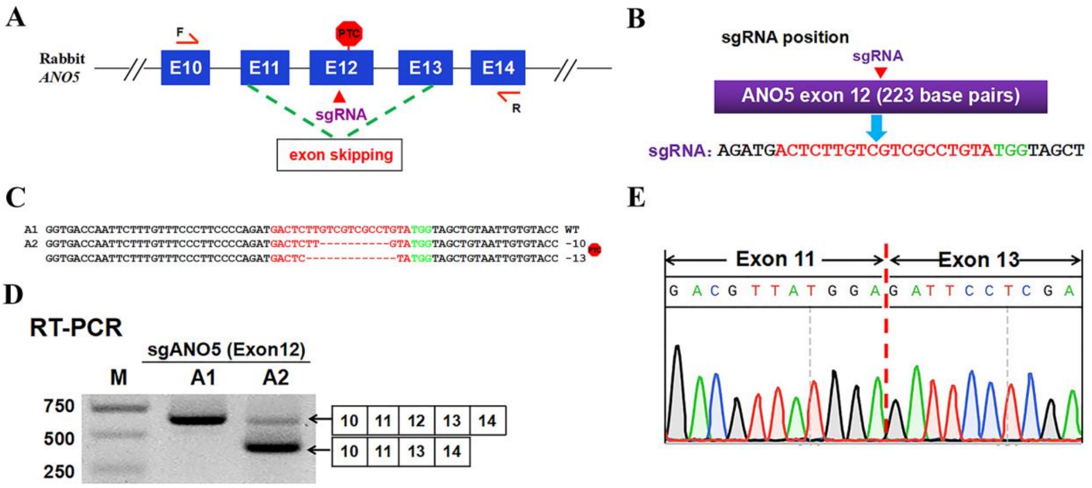

Figure S3: PTC mutation in exon 12 of *ANO5* gene induces exon skipping. (A) Schematic of sgRNA target site in exon 12 of the rabbit *ANO5* gene. Blue boxes are represented exons of *ANO5*. The target site of sgRNAs is indicated in red triangle; The PTC site is indicated by red octagon. (B) sgRNA target sites in exon 12 of *ANO5* gene. (C) Sanger sequencing results of the *ANO5* PCR product. (D) RT-PCR analysis of *ANO5* gene-editing rabbits for exons 10, 11, 12, 13 and 14. Gel images have been cropped. M, which shows the DL2000 ladder, indicates band size. A1-A2, the *ANO5* gene-edited rabbits used in this study. (E) Sequence analysis of cDNA bands confirmed the splicing of exon 12.

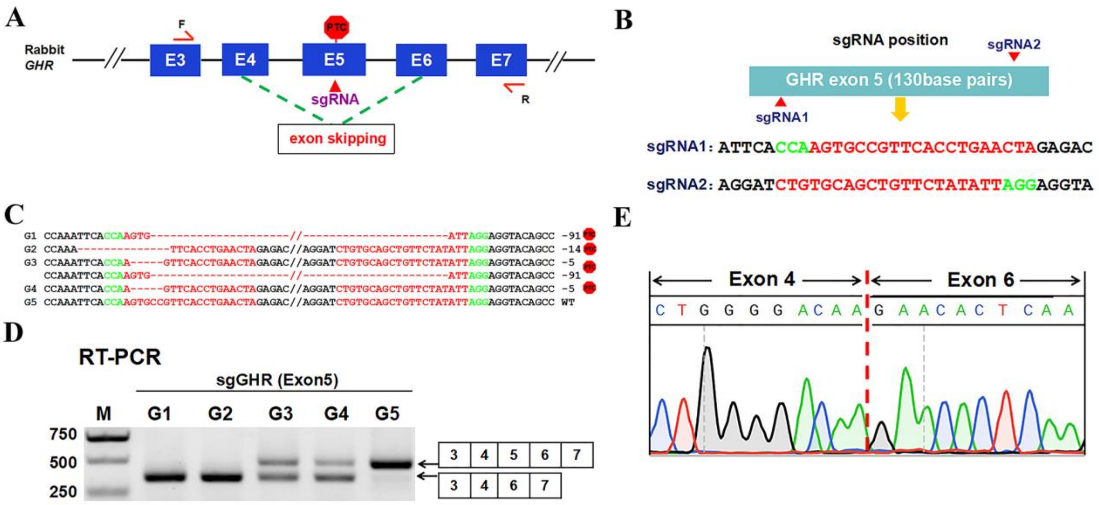

Figure S4: PTC mutation in exon 5 of *GHR* gene induces exon skipping. (A) Schematic diagram of sgRNA target site in exon 5 of the rabbit *GHR* locus. Exons are indicated by blue boxes. The target site of sgRNAs is indicated in red triangle; The PTC site is indicated by red octagon. (B) sgRNA target sites in *GHR* exon 5. (C) Sanger sequencing results for the *GHR* PCR product. (D) RT-PCR analysis of *GHR* gene-editing rabbits for exons 3, 4, 5, 6 and 7. Gel images have been cropped. M, which shows the DL2000 ladder, indicates band size. G1-G5, the *GHR* gene-edited rabbits used in this study. (E) Sequence analysis of cDNA bands confirmed the splicing of exon 5.

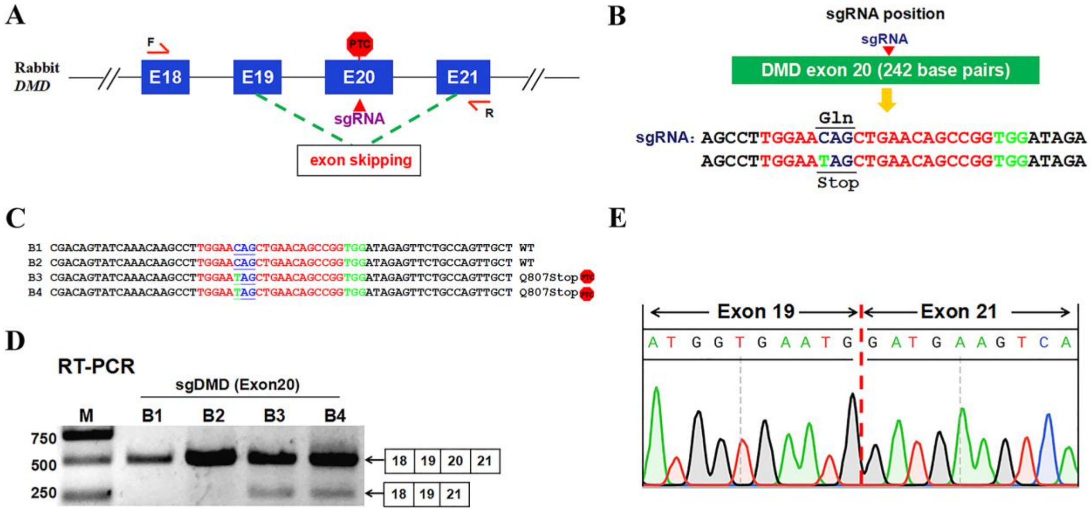

Figure S5: PTC mutation in exon 20 of *DMD* gene induces exon skipping. (A) Schematic diagram of sgRNA target site in exon 20 of the rabbit *DMD* locus. Blue boxes are represented exons of *DMD*. The target site of sgRNAs is indicated in red triangle; The PTC site is indicated by red octagon. (B) sgRNA target sites in exon 20 of *DMD*. (C) Sanger sequencing results for the

DMD PCR product. (D) RT-PCR analysis of *DMD* base editing rabbits for exons 18, 19, 20 and 21. Gel images have been cropped. M, which shows the DL2000 ladder, indicates band size. B1-B4, the *DMD* gene-edited rabbits used in this study. (E) Sequence analysis of cDNA bands confirmed the splicing of exon 20.

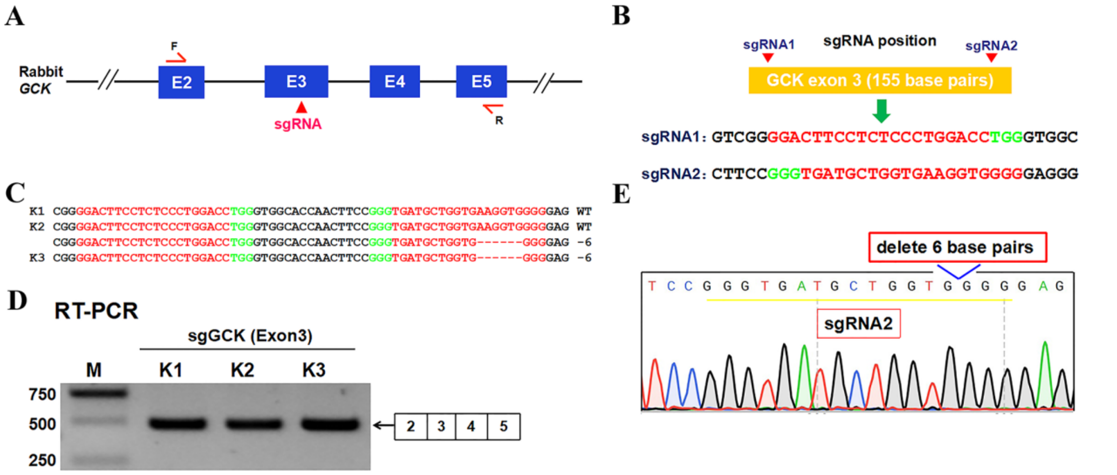

Figure S6: Non-frame shift mutation in exon 6 of *GCK* did not induce exon skipping. (A) Schematic diagram of sgRNA target site in exon 3 of the rabbit *GCK* gene locus. Exons are indicated by blue boxes. The target site of sgRNAs is indicated in red triangle; The PTC site is indicated by red octagon. (B) sgRNA target sites in exon 3 of the *GCK* gene. (C) Sanger sequencing results of the *GCK* PCR product. (D) RT-PCR analysis of *GCK* gene-editing rabbits for exons 2, 3, 4 and 5. Gel images have been cropped. M, which shows the DL2000 ladder, indicates band size. K1, K2, K3, the *GCK* gene-edited rabbits used in this study. (E) Sanger sequencing of the RT-PCR products did not reveal exon skipping.

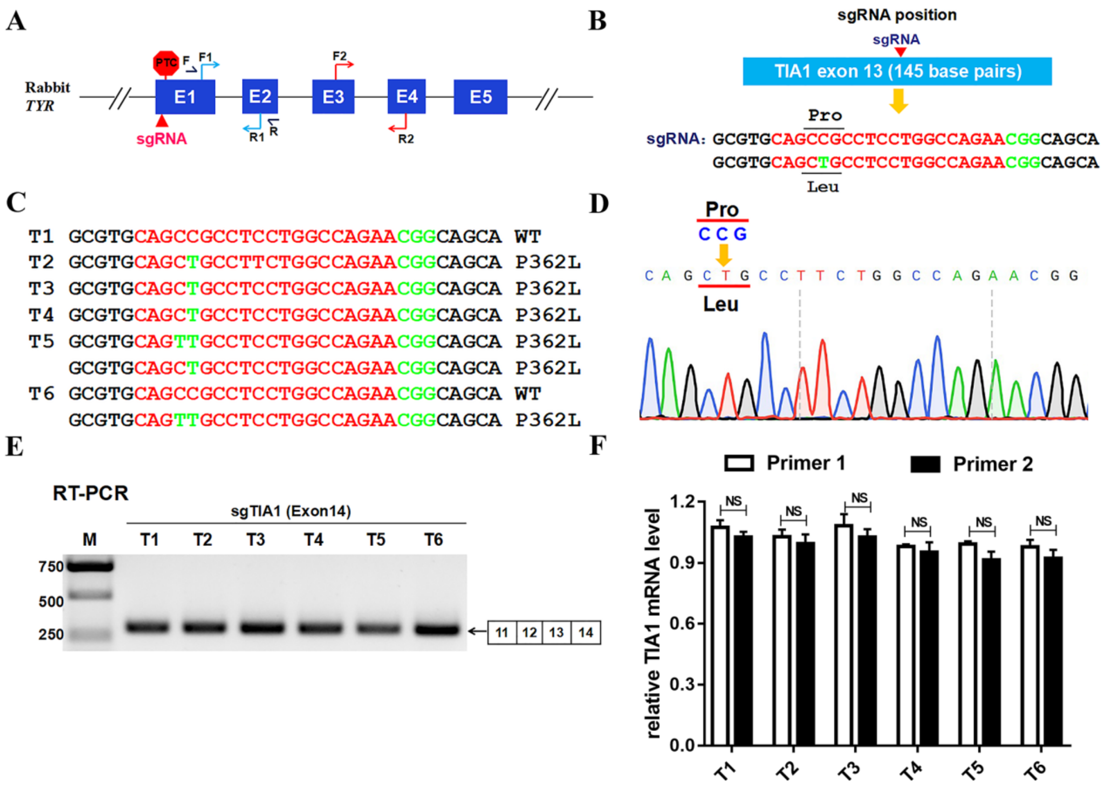

Figure S7: Missense mutations in the last exon of the *TIA1* gene did not induce exon skipping. (A) Schematic diagram of sgRNA target site in exon 14 of the rabbit *TIA1* gene locus. Exons are indicated by blue boxes. The target site of sgRNAs is indicated in red triangle; The PTC site is indicated by red octagon. (B) sgRNA target sites in exon 14 of the *TIA1* gene. (C) Genomic PCR from a rabbit showing changes from C to T. (D) RT-PCR analysis of *TIA1* gene-editing rabbits for exons 11, 12, 13 and 14. Gel images have been cropped. M, which shows the DL2000 ladder, indicates band size. (E) Sanger sequencing of the RT-PCR products did not reveal exon skipping. Gel images have been cropped. M, which shows the DL2000 ladder, indicates band size. T1-T5, the *TIA1* gene-edited rabbits used in this study. (F) qPCR studies revealed that the transcript levels of different exons did not significantly differ. NS, no significantly different. Primer 1, the primer of *TIA1*-F1 and *TIA1*-R1 were used for qPCR. Primer 2, the primer of *TIA1*-F2 and *TIA1*-R2 were used for qPCR.

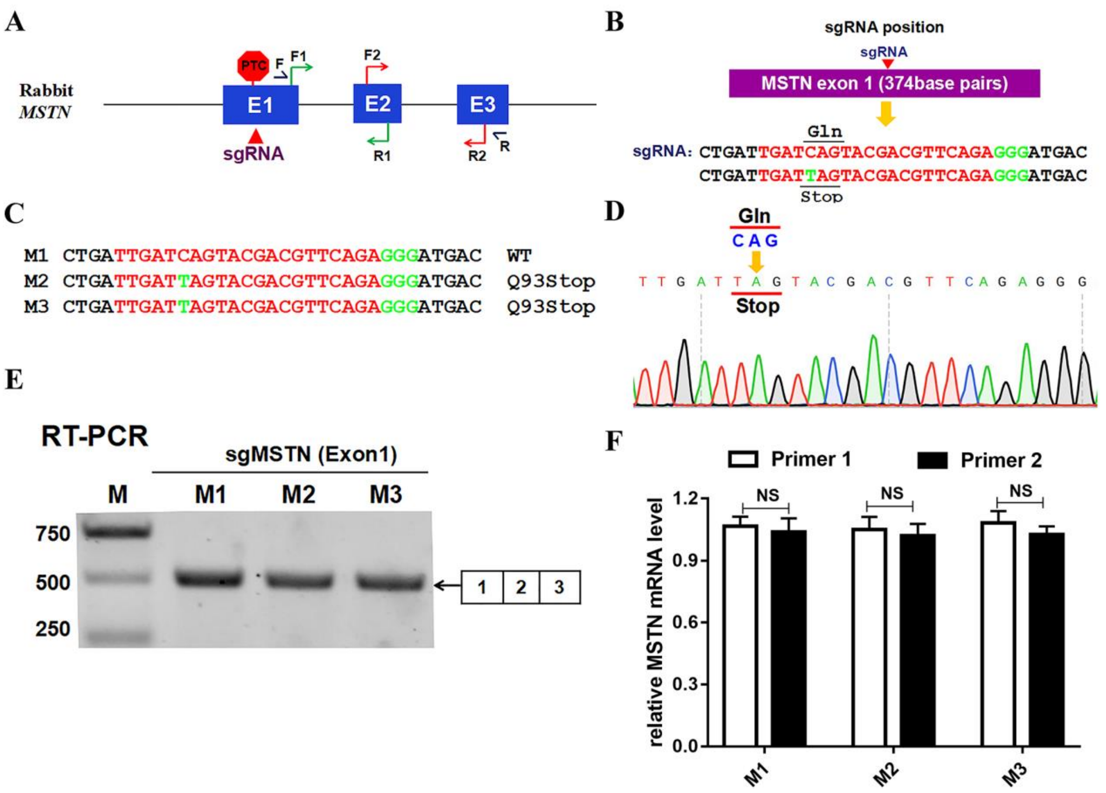

Figure S8: PTCs mutation in exon 1 of *MSTN* gene did not induce exon skipping. (A) Schematic diagram of sgRNA target site in exon 1 of the rabbit *MSTN* gene locus. Exons are indicated by blue boxes. The target site of sgRNAs is indicated in red triangle; The PTC site is indicated by red octagon. (B) sgRNA target sites in exon 1 of the *MSTN* gene. (C) Genomic PCR from a rabbit showing changes from C to T. (D) Sanger sequencing of the RT-PCR products did not reveal exon skipping. (E) RT-PCR analysis of *MSTN* gene editing rabbits for exons 1, 2 and 3. Gel images have been cropped. M, which shows the DL2000 ladder, indicates band size. Gel images have been cropped. M, which shows the DL2000 ladder, indicates band size. M1, M2, M3, the *MSTN* gene-edited rabbits used in this study. (F) The transcript levels of different exons using exon specific primer by qPCR. NS, no significantly different. Primer 1, the primer of *MSTN*-F1 and *MSTN*-R1 were used for qPCR. Primer 2, the primer of *MSTN*-F2 and *MSTN*-R2 were used for qPCR.

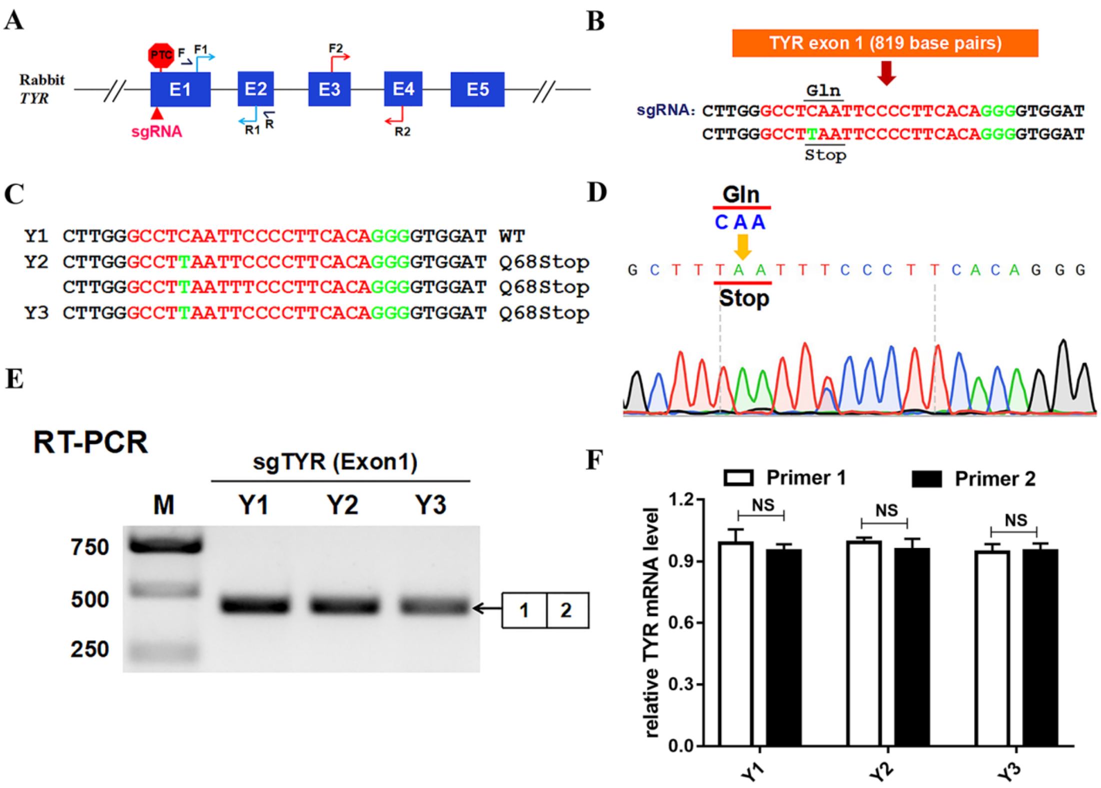

Figure S9: PTCs in exon 1 of *TYR* gene did not induce exon skipping. (A) Schematic diagram of sgRNA target site in exon 1 of the rabbit *TYR* gene locus. Exons are indicated by blue boxes. The target site of sgRNAs is indicated in red triangle; The PTC site is indicated by red octagon. (B) sgRNA target sites in exon 1 of the *TYR* gene. (C) Genomic PCR from a rabbit showing changes from C to T. (D) Sanger sequencing of the RT-PCR products did not reveal exon skipping. (E) RT-PCR analysis of *TYR* gene editing rabbits for exons 1, 2 and 3. Gel images have been cropped. M, which shows the DL2000 ladder, indicates band size. Y1, Y2, Y3, the *TYR* gene-edited rabbits used in this study. (F) qPCR studies revealed that the transcript levels of different exons did not significantly differ. NS, no significantly different. Primer 1, the primer of *TYR* -F1 and *TYR* -R1 were used for qPCR. Primer 2, the primer of *TYR* -F2 and *TYR* -R2 were used for qPCR.

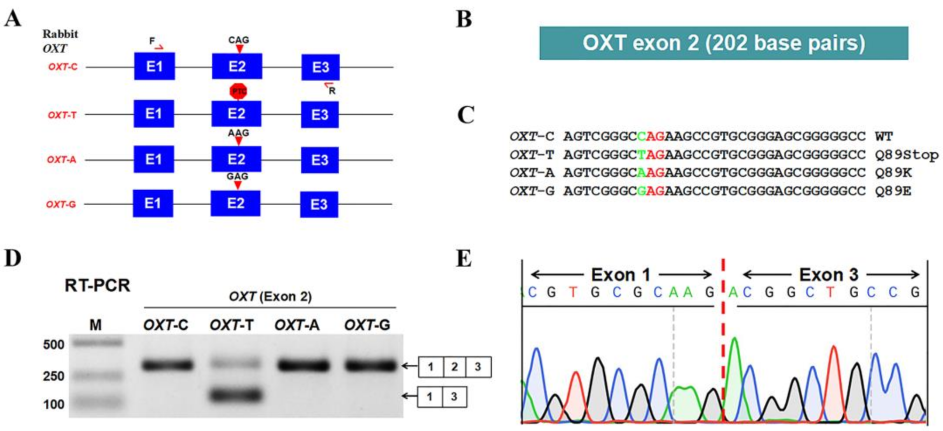

Figure S10: PTC mutation in exon 2 of *OXT* gene induces exon skipping. (A) Schematic diagram of the PTC mutation and when T in PTC was replaced by G or A in exon 2 of the rabbit *OXT* locus. Blue boxes are represented exons of rabbit ***OXT***. The PTC site is indicated by red octagon. The F and R primers were used for genotype and exon skipping determination. *OXT*-C, WT. *OXT*-T, PTC mutation. *OXT*-A, C-A mutation. *OXT*-C, C-G mutation. (B) Sanger sequencing results for the *OXT* PCR product. (C) RT-PCR analysis of exon skipping in transient system when T in PTC replaced by G and A. Gel images has been cropped. M, which shows the DL2000 ladder, indicates band size. (D) Sequence analysis of cDNA bands confirmed the splicing of exon 2 in PTC mutation, while not determined in missense mutation of rabbit *OXT* gene.
