# Supplementary material for: CRISPR-induced exon skipping is dependent on premature termination codon mutations
Source: Genome Biol. 2018 Oct 17;19:164. doi: 10.1186/s13059-018-1532-z (PMC6193291; doi:10.1186/s13059-018-1532-z)
Supplement: Supplementary file 3 — Table S1. Examples according to the published reports on CRISPR/Cas9-induce exon-skipping and the case reports in clinical. Table S2. PCR primers for genotyping of the gene editing rabbits. Table S3. Primers for RT-PCR analysis of exon skipping. Table S4. Primers for qPCR analysis. (PDF 561 kb) [file 13059_2018_1532_MOESM3_ESM.pdf]

Supplemental Tables

Table S1. Examples according to the published reports on CRISPR/Cas9-induce exon-skipping and the case reports in clinical.

Reference	Gene (Gene bank)	Method	Nature of mutation	PTC in Exon	Exon skipping
[1]	*KRAS* (NM_021284.6)	CRISPR/Cas9	-2bp in exon 2	Exon 2	Exon 2
			-1bp in exon 2	No	No
	Ctnnb1 (NM_001098209.1)	CRISPR/Cas9	-3bp in exon 3	No	No
			+1bp in exon 3	Exon 3	Exon 3
			+1bp in exon 3	Exon 3	Exon 3, 4
	P65 (NM_001365067.1)	CRISPR/Cas9	+1bp in exon 6	No	No
[2]	*DMD* (NM_000109.3)	CRISPR/Cas9	-18bp in exon 45	exon 45	exon 45
			+1bp in exon 45	exon 45	exon 45
			-10bp in exon 45	No	No
			-8bp in exon 45	No	No
			-7bp in exon 45	No	No
			-1bp in exon 45	No	No
[3]	*Creb3l3a* (NM_001020673.1)	CRISPR/Cas9	g.357C>T in exon 2	Exon 2	Exon 2
	Pla2g12b (NM_213430.1)	CRISPR/Cas9	g.10194A>T in exon 4	No	No
	Smyd1a (NM_205540.2)	CRISPR/Cas9	-7bp in exon 3	Exon 3	Exon 3
			-13bp in exon 2	Exon 2	Exon 2
			-40bp in exon 2	Exon 2	Exon 2
[4]	*PHACTR1* (NM_001242648.2)	CRISPR/Cas9	+4bp in exon 8	Exon 8	Exon 8
			-1bp in exon 8	Exon 8	Exon 8
			-1bp in exon 9	Exon 9	Exon 9
			+22 bp in exon 10	Exon 10	Exon 10
	Adgrl4 (NM_213367.2)	TALEN	-5bp in exon 2	Exon 2	Exon 2
	LGALS8 (NM_006499.4)	TALEN	p.Leu212Ter in exon 9	Exon 9	Exon 9
[5]	*BRCA1* (NM_007294.3)	Case report	p. Glu1694Ter in exon 18	Exon 18	Exon 18
[6]	*FBN1* (NM_000138.4)	Case report	PTC mutations in exon 51	Exon 51	Exon 51
[7]	*CEP290* (NM_025114.3)	Case report	c.1666del in exon 17	Exon 18	Exon 18
			c.508A>T in exon 8	Exon 8	Exon 8
			c. 4090G>T in exon 32	Exon 32	Exon 32
[8]	*NF1* (NM_000267.3)	Case report.	c.4367+1G>C in exon 32	Exon 32	Exon 32
[9]	*LAMB3* (NM_000228.2)	Case report	c.1903C>T in exon 14	Exon 14	Exon 14
[10]	*LAMB3* (NM_000228.2)	Case report	c.1978C>T in exon 15	Exon 15	Exon 15
[11]	*LAMC2* (NM_005562.2)	Case report	c.1045C>T in exon 8	Exon 8	Exon 8
[12]	*LAMA3* (NM_000227.4)	Case report	c.3928A>T in exon 30	Exon 30	Exon 30
[13]	*LAMA2* (NM_000426.3)	Case report	p. Arg744Stop in exon 15	Exon 15	Exon 15

[14]	*COL11A2* (NM_001163771.1)	Case report	p. Arg893Stop in exon 57	Exon 57	Exon 57
[15]	*CFTR* (NM_000492.3)	Case report	p. Arg553X in exon 11	Exon 11	Exon 11
[16]	*AAAS* (NM_001173466.1)	Case report	c.1331+1G>A in exon 14	Exon 14	Exon 14
[17]	*DOCK9* (NM_001130048.1)	Case report	c. 2262A>C in exon 20	Exon 20	Exon 20
[18]	*CASC5* (NM_144508.4)	Case report	c. 6673-19T>A in exon 25	Exon 25	Exon 25
[19]	*NF1* (NM_000267.3)	Case report	p. Trp336X in exon 7<br>p. Gln315X in exon 7<br>p. Arg304X in exon 7	Exon 7<br>Exon 7<br>Exon 7	Exon 7<br>Exon 7<br>Exon 7
[20]	*PS* (NM_000267.3)	Case report	p. Ser62Stop in exon 4	Exon 4	Exon 4
[21]	*ACY1* (NM_000666.2)	Case report	c.1001+5del6 in exon 13	Exon 13	Exon 13
[22]	*CYP27B1* (NM_000785.3)	Case report	c.1022-1037del16: p in exon 7<br>c. 934_935delAC in exon 7<br>c.1215 T>C in exon 7	Exon 7<br>Exon 7<br>No	Exon 7<br>Exon 7<br>No
[23]	*FANCM* (NM_001308133.1)	Case report	c.5791C>T in exon 22	Exon 22	Exon 22

Reference:

1. Mou H, Smith JL, Peng L, Yin H, Moore J, Zhang XO, Song CQ, Sheel A, Wu Q, Ozata DM, et al: **CRISPR/Cas9-mediated genome editing induces exon skipping by alternative splicing or exon deletion.** *Genome Biol* 2017, **18:**108.
2. Li HL, Fujimoto N, Sasakawa N, Shirai S, Ohkame T, Sakuma T, Tanaka M, Amano N, Watanabe A, Sakurai H, et al: **Precise correction of the dystrophin gene in duchenne muscular dystrophy patient induced pluripotent stem cells by TALEN and CRISPR-Cas9.** *Stem Cell Reports* 2015, **4:**143-154.
3. Anderson JL, Mulligan TS, Shen MC, Wang H, Scahill CM, Tan FJ, Du SJ, Busch-Nentwich EM, Farber SA: **mRNA processing in mutant zebrafish lines generated by chemical and CRISPR-mediated mutagenesis produces unexpected transcripts that escape nonsense-mediated decay.** *PLoS Genet* 2017, **13:**e1007105.
4. Lalonde S, Stone OA, Lessard S, Lavertu A, Desjardins J, Beaudoin M, Rivas M, Stainier DYR, Lettre G: **Frameshift indels introduced by genome editing can lead to in-frame exon skipping.** *PLoS One* 2017, **12:**e0178700.
5. Liu HX, Cartegni L, Zhang MQ, Krainer AR: **A mechanism for exon skipping caused by nonsense or missense mutations in BRCA1 and other genes.** *Nat Genet* 2001, **27:**55-58.
6. Dietz HC, Valle D, Francomano CA, Kendzior RJ, Jr., Pyeritz RE, Cutting GR: **The skipping of constitutive exons in vivo induced by nonsense mutations.** *Science* 1993,

259:680-683.
7. Barny I, Perrault I, Michel C, Soussan M, Goudin N, Rio M, Thomas S, Attie-Bitach T, Hamel C, Dollfus H, et al: **Basal exon skipping and nonsense-associated altered splicing allows bypassing complete CEP290 loss-of-function in individuals with unusually mild retinal disease.** *Hum Mol Genet* 2018.
8. Fu Y, Zhang JQ, Jiang CL, Wang HY: **Identification of a missense mutation causing exon skipping in a neurofibromatosis type 1 patient.** *J Dermatol* 2018.
9. Pulkkinen L, Christiano AM, Gerecke D, Wagman DW, Burgeson RE, Pittelkow MR, Uitto J: **A homozygous nonsense mutation in the beta 3 chain gene of laminin 5 (LAMB3) in Herlitz junctional epidermolysis bullosa.** *Genomics* 1994, **24:**357-360.
10. Kivirikko S, McGrath JA, Pulkkinen L, Uitto J, Christiano AM: **Mutational hotspots in the LAMB3 gene in the lethal (Herlitz) type of junctional epidermolysis bullosa.** *Hum Mol Genet* 1996, **5:**231-237.
11. Takizawa Y, Shimizu H, Pulkkinen L, Nonaka S, Kubo T, Kado Y, Nishikawa T, Uitto J: **Novel premature termination codon mutations in the laminin gamma2-chain gene (LAMC2) in Herlitz junctional epidermolysis bullosa.** *J Invest Dermatol* 1998, **111:**1233-1234.
12. Muhle C, Jiang QJ, Charlesworth A, Bruckner-Tuderman L, Meneguzzi G, Schneider H: **Novel and recurrent mutations in the laminin-5 genes causing lethal junctional epidermolysis bullosa: molecular basis and clinical course of Herlitz disease.** *Hum Genet* 2005, **116:**33-42.
13. Di Blasi C, He Y, Morandi L, Cornelio F, Guicheney P, Mora M: **Mild muscular dystrophy due to a nonsense mutation in the LAMA2 gene resulting in exon skipping.** *Brain* 2001, **124:**698-704.
14. Vuoristo MM, Pappas JG, Jansen V, Ala-Kokko L: **A stop codon mutation in COL11A2 induces exon skipping and leads to non-ocular Stickler syndrome.** *Am J Med Genet A* 2004, **130A:**160-164.
15. Hull J, Shackleton S, Harris A: **The stop mutation R553X in the CFTR gene results in exon skipping.** *Genomics* 1994, **19:**362-364.
16. Kallabi F, Ben Rhouma B, Baklouti S, Ghorbel R, Felhi R, Keskes L, Kamoun H: **Splicing Defects in the AAAS Gene Leading to both Exon Skipping and Partial Intron Retention in a Tunisian Patient with Allgrove Syndrome.** *Horm Res Paediatr* 2016, **86:**90-93.
17. Karolak JA, Rydzanicz M, Ginter-Matuszewska B, Pitarque JA, Molinari A, Bejjani BA, Gajecka M: **Variant c.2262A>C in DOCK9 Leads to Exon Skipping in Keratoconus Family.** *Invest Ophthalmol Vis Sci* 2015, **56:**7687-7690.
18. Szczepanski S, Hussain MS, Sur I, Altmuller J, Thiele H, Abdullah U, Waseem SS, Moawia A, Nurnberg G, Noegel AA, et al: **A novel homozygous splicing mutation of CASC5 causes primary microcephaly in a large Pakistani family.** *Hum Genet* 2016, **135:**157-170.
19. Wimmer K, Eckart M, Stadler PF, Rehder H, Fonatsch C: **Three different premature stop codons lead to skipping of exon 7 in neurofibromatosis type I patients.** *Hum Mutat* 2000, **16:**90-91.
20. Okamoto Y, Yamazaki T, Katsumi A, Kojima T, Takamatsu J, Nishida M, Saito H: **A novel**

nonsense mutation associated with an exon skipping in a patient with hereditary protein S deficiency type I. *Thromb Haemost* 1996, **75:**877-882.

21. Ferri L, Funghini S, Fioravanti A, Biondi EG, la Marca G, Guerrini R, Donati MA, Morrone A: **Aminoacylase I deficiency due to ACY1 mRNA exon skipping.** *Clin Genet* 2014, **86:**367-372.

22. Demir K, Kattan WE, Zou M, Durmaz E, BinEssa H, Nalbantoglu O, Al-Rijjal RA, Meyer B, Ozkan B, Shi Y: **Novel CYP27B1 Gene Mutations in Patients with Vitamin D-Dependent Rickets Type 1A.** *PLoS One* 2015, **10:**e0131376.

23. Peterlongo P, Catucci I, Colombo M, Caleca L, Mucaki E, Bogliolo M, Marin M, Damiola F, Bernard L, Pensotti V, et al: **FANCM c.5791C>T nonsense mutation (rs144567652) induces exon skipping, affects DNA repair activity and is a familial breast cancer risk factor.** *Hum Mol Genet* 2015, **24:**5345-5355.

Table S2. PCR primers for genotyping of the gene editing rabbits

Name	Primers	Sequence(5’-3’)	Produce size (bp)
DMD	*DMD*-51-F	TAGTTTGGCTCAGATTGTAG	507
	DMD-51-R	AGAATAGACAAAGCAGTGTG	
ANO5	*ANO5*-F	CCCATATGCCTTGTTCTATT	492
	ANO5-R	GCATGATTAGGAACCCTTT	
LMNA	*LMNA*-F	GAAGGGTGGAGAGACAGGAA	490
	LMNA-R	GCTACATCCAATGAGTGAAAGA	
GHR	*GHR*-F	CATTGGTGTCACCTCCTAGATAC	713
	GHR-R	CACACTCACACTCATCCATACA	
DMD	*DMD*-20-F	TCTTTCAGCCTGTGACTTCAG	421
	DMD-20-R	GTGGCTTAGCTAAATCTGTAGGA	
GCK	*GCK*-F	GTGCCCCAGTCCACCATGGAG	626
	GCK-R	CCAACAGCCTCTGCCGGGTTG	
MSTN	*MSTN*-F	GGCCCAGTGGATCTAAATGAA	449
	MSTN-R	AGACTGTCTTTCCTGCTTCTTAC	
TIA1	*TIA1*-F	GGCATTACTGTTACGTTGGTATTT	459
	TIA1-R	GGCAGACATCCAGCATCTT	
TYR	*TYR*-F	ATCCGCTCAAGCAGGTATTG	454
	TYR-R	AGTGAGGTAGGCAAGGAATTTG	
OXT	*OXT-F*	GCAAGGTGAGTGTTCACAGG	609
	OXT-R	TATTATTCCTGGGAGTGGCTGA	

DMD-51, the primer used for Fig.S1. *DMD*-20, the primer used for Fig.S5. *OXT,* the primer used for Fig.S10 .

Table S3. Primers for RT-PCR analysis of exon skipping.

Name	Primers	Sequence(5’-3’)	Produce size (bp)
DMD	*DMD*-51-F	GTCAACTATCTACTGCAAGAGC	459
	DMD-51-R	CTGTACTTCATCCCACTGATTC	
ANO5	*ANO5*-F	CGACAAGCCACATTGGAATAC	610
	ANO5-R	ACTGAGTCGTCAGTTCGATTAG	
LMNA	*LMNA*-F	CATCAAGGCCGCCTACGAG	418
	LMNA-R	CAGCTCCTCGCTGTAGATGTT	
GHR	*GHR*-F	CACTAGCAGGGTCAAGTGATG	478
	GHR-R	TCTGCATGAATCCCGGTTAAG	
DMD	*DMD*-20-F	TGGTGGAACAGATGGTGAATG	492
	DMD-20-R	TGGTACTGATCGTCTCCTGATAG	
GCK	*GCK*-F	GAGGACCTGAAGAAGGTGATG	466
	GCK-R	CCACGATGTTGTTCCCTTCA	
MSTN	*MSTN*-F	TTATCACGCTACGACGGAAAC	494
	MSTN-R	GCTCATCACAGTCAAGACCAA	
TYR	*TYR*-F	ACTACGAGCCCAGACTATGT	432
	TYR-R	GCTCTGTCGGCTATTGTACTC	
TIA1	*TIA1*-F	GTTCGGTTCAACTCCCATGA	291
	TIA1-R	GGAAGACTGCGTCTGGTTAAA	
OXT	*OXT*-F	GCTGACCTCGGCCTGCTACAT	331
	OXT-R	GCGCTCGGAGAAGGCGGCCTCG	

DMD-51, the primer used for Fig.S1. *DMD*-20, the primer used for Fig.S5. *OXT,* the primer used for Fig.S10.

Table S4. Primers for qPCR analysis.

Name	Primers	Sequence(5’-3’)	Produce size (bp)
MSTN	*MSTN*-F1	GCTCTTTGGAAGATGACGATTA	109
	MSTN-R1	AAGAAGCAACATTTGGGTTT	
	MSTN-F2	CCTGAGGCTCATCAAACCTATG	106
	MSTN-R2	CACATCAATGCTCTGCCAAATAC	
TYR	*TYR*-F1	GCTGTGATGTTTGTACAGATGAG	113
	TYR-R1	CTCCAACCGGCTACAGATAAT	
	TYR-F2	CCATGCGTTTGTTGACAGTATT	111
	TYR-F2	ATGTAGGATTCCCGGTTGTG	
TIA1	*TIA1*-F1	CACAACAGATTGGCCAGTACAT	116
	TIA1-R1	CATCCACGGTGCGGAAGAC	
	TIA1-F2	CGAAGGGATATGGCTTTGTCT	112
	TIA1-F2	GTTGCCCAGTTAGTTCTGATTTG	
